# An unusual case of chest wall glomus tumor presenting with axillary pain: a case report and literature review

**DOI:** 10.1186/s40001-021-00518-6

**Published:** 2021-05-25

**Authors:** Leila Oryadi Zanjani, Bahman Shafiee Nia, Farzad Vosoughi, Elham Mirzaian, Leila Aghaghazvini, Aidin Arabzadeh

**Affiliations:** 1 Orthopedic Surgery Department, Shariati Hospital, Tehran University of Medical Sciences, Tehran, Iran; 2 Joint Reconstruction Research Center, Orthopedic Surgery Department, End of Keshavarz Blvd, Imam Khomeini Hospital, Tehran University of Medical Sciences, 1419733141 Tehran, Iran; 3 Department of Pathology, Shariati Hospital, Tehran University of Medical Sciences, Tehran, Iran; 4 Department of Radiology, Shariati Hospital, Tehran University of Medical Sciences, Tehran, Iran

**Keywords:** Glomus tumor, Chest wall, Case report

## Abstract

**Background:**

Glomus tumor is an uncommon soft tissue tumor. However, as the tumor causes significant disability, its early diagnosis is essential. It involves subungual areas of fingers and toes in most cases, and its extra-digital involvement is rarely seen. To the best of the authors' knowledge, only a few chest wall involvement cases have been reported in the literature.

**Case presentation:**

In this paper, we describe a 63-year-old patient with a chest wall glomus tumor presenting with axillary paroxysmal pain and limitation in his shoulder range of motion that had been missed for nearly 15 years. His symptoms were relieved immediately following surgical excision.

**Conclusion:**

Glomus tumors may involve any part of the human body. It is curable with surgical excision in most cases. Therefore, a correct early diagnosis has paramount importance. A high index of suspicion is needed for early diagnosis, especially when the tumor involves uncommon anatomic areas.

## Background

Glomus tumor (GT) is a mesenchymal neoplasm that originates from modified smooth muscle cells of the glomus body known as glomus cells [1]. Less than 2 percent of soft tissue tumors turn out to be GT [2]. It causes severe paroxysmal pain crises, point tenderness, and temperature sensitivity, leading to significant functional impairment. Due to the low incidence of the tumor and its various manifestations, many of the affected patients are missed, and their pain is attributed to a mental problem [3].

The lesion is mostly benign, and its excision has a cure rate of around 90 percent [4]. However, malignant [5–9] and even lethal varieties [8, 10] also are described in the literature.

The tumor mostly involves the hand and foot digits especially the subungual area [11]. However, GT may affect any part of the human body. Regarding extra-digital GT, 60 percent of cases are reported to affect the upper extremity, and only 24 percent are seen to involve trunk area [4]. To the best of our knowledge, 7 cases of chest wall GT are reported in the literature.

In this article, we report a case of chest wall GT with an uncommon presentation of chronic axillary pain. This article highlights a precise history taking and a high index of suspicion to recognize an unusual presentation of GT. We obtained informed consent from our patient for the publication of this case report.

## Case presentation

The patient was a 63-year-old male who presented to our clinic with left axillary pain and significant shoulder movement limitation. His symptoms started 15 years ago and did not subside despite using several pain killers. Initially, his pain was vague and sensed over the entire left shoulder. However, after 7 years, his pain changed in nature to a paroxysmal and lancinating one localized to the left axilla. He did not state any history of trauma. He had undergone radiologic and cardiologic work-ups several times without achieving a definite diagnosis. From 6 years ago, he noticed an axillary lump, assuming it to be an insignificant lesion unrelated to his chief complaint. According to the patient, the mass was sensitive to cold temperature and obliged him to wear warm clothes. As the mass size became larger and the pain was exacerbated, he became worried and came to our clinic for further evaluation.

Upon physical examination, his lump was revealed to be a fixed and painful mass located at the patient's left chest wall (in the mid-axillary line). No enlarged axillary lymph nodes were detected. His left shoulder's passive motion was in full range, but his active range of motion, especially active forward elevation, was limited.

**Fig. 1:**
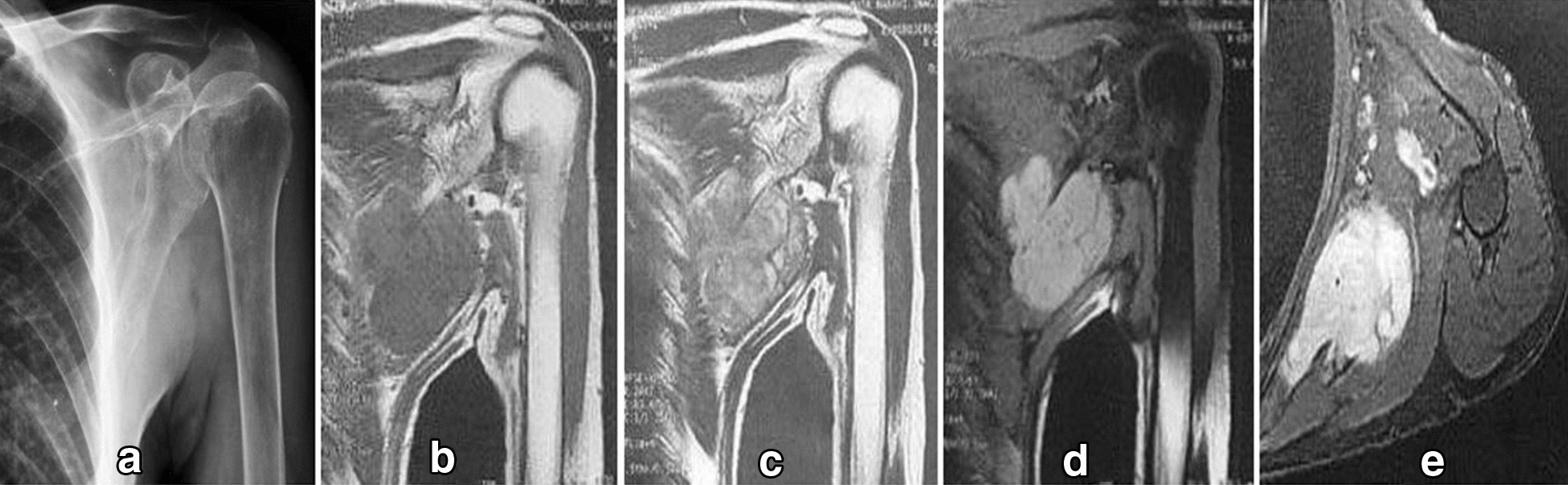
Diagnostic imaging. **a** Radiograph shows a soft tissue lesion affecting chest wall in the infra-axillary region. **b**–**e**, MRI shows a lobulated mass iso-signal in T1W and heterogeneous to high signal in T2W and high signal in fat-saturated sequences with strong post-contrast enhancement in the infra-axillary intermuscular fat plane. The tumor had close contact with adjacent vessels

The chest X-ray and shoulder X-ray of our patient were insignificant. His left shoulder magnetic resonance imaging (MRI) revealed a 5-cm (in the largest diameter) left axillary soft tissue mass at the mid-axillary line. The tumor was deep to the fascia and over the posterolateral chest wall extending from third to fifth ribs with no visible rib cage involvement [Fig. 1].

Our differential diagnoses were schwannoma, hemangioma, glomus tumor, lipoma, and their malignant counterparts. Thus, we decided to perform an open biopsy. The histopathology report was in favor of a GT. At the next step, the excision of the mass was planned. We approached the tumor through the mid-axillary longitudinal incision. The previous incision for biopsy was included in the surgical approach. During the operation, the lesion was observed to be an encapsulated mass situated between the scapular body and chest wall. There was no gross adhesion between the tumor and the surrounding tissues. Therefore, it was separated by blunt dissection. After ligating its main vascular pedicle, the mass was excised completely and sent for histopathologic assessment.

**Fig. 2:**
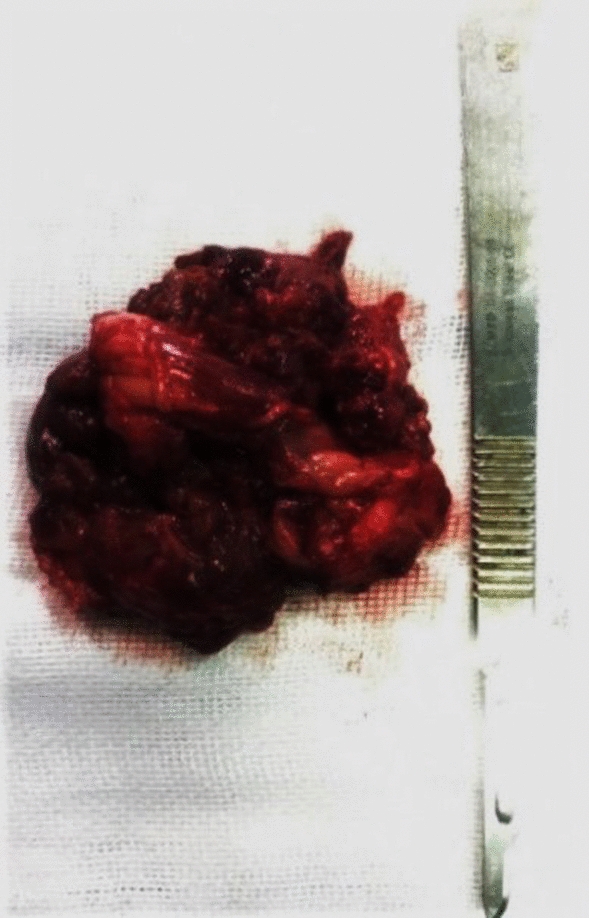
Macroscopic appearance of tumor

Macroscopically the lesion had an irregular red–purple tissue and sized 5 × 4 × 2 cm [Fig. 2].

**Fig. 3:**
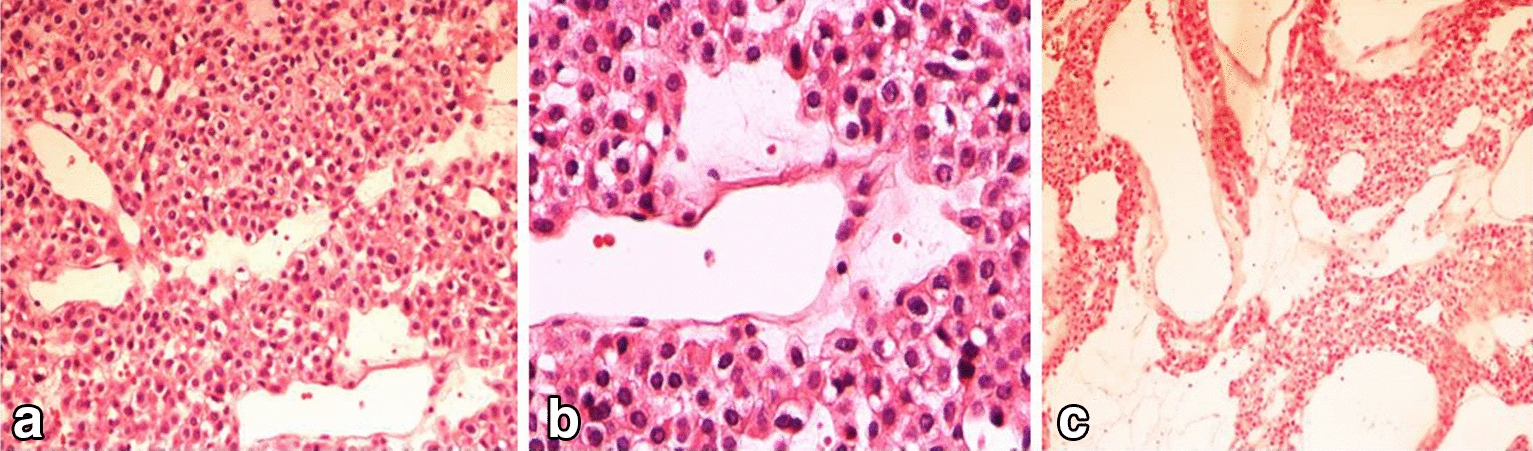
Microscopic appearance. **a** Thin-walled blood vessels surrounded by a solid proliferation of round and epithelioid shape cells with round nuclei and lightly acidophilic cytoplasm (H&M,20×). **b** No significant nuclear atypia or mitosis was identified (H&M,40×). **c**, Cavernous hemangioma-like vascular structures surrounded by clusters of glomus cells (H&E 10 ×)

Microscopic examination showed vascular structures surrounded by clusters of glomus cells with no significant nuclear atypia or mitosis. These features could be suggestive of glomangioma, a variant of GT [Fig. 3].

**Fig. 4:**
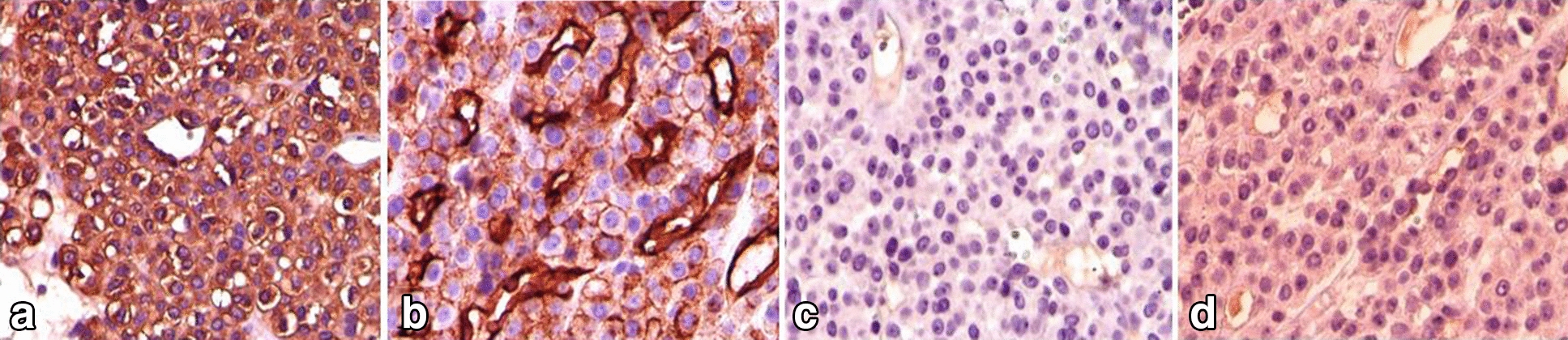
Immunohistochemical staining. **a**, **b** SMA, and CD34 positivity. **c**, **d** CK, and desmin negativity

The tumor cells were positive for SMA and CD34 and negative for desmin and cytokeratin on immunohistochemical study [Fig. 4].

The pain and sensitivity to cold disappeared immediately after the surgery. We followed the patient 1-month post-surgery and every 3 months afterward. At the final follow-up, 19 months after surgery, no recurrence was detected. The patient was satisfied and had no pain and sensitivity to coldness.

## Discussion

**Fig. 5:**
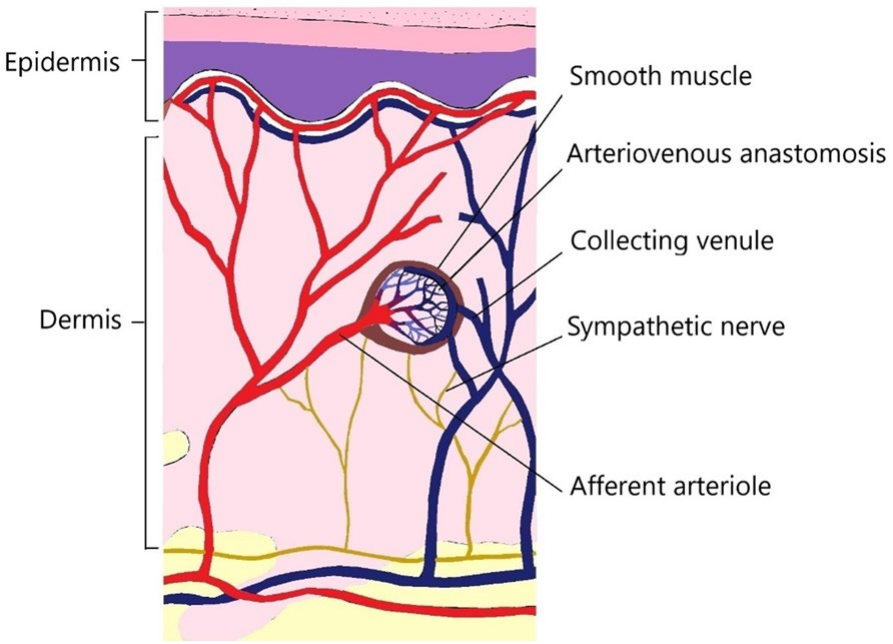
A schematic view of the glomus body in the dermis layer of the skin

Glomus body (glomus in Latin means ball) is a specific structure in the dermis of the skin regulating the body's temperature and blood pressure. It consists of a peripheral capsule, afferent arterioles, collecting venules, arteriovenous anastomoses known as Sucquet–Hoyer canal, nerve fibers, and modified smooth muscle cells named glomus cells [Fig. 5] [2, 12]. The pathologic proliferation of the glomus cells either due to an underlying hamartoma or as a reaction to previous trauma may lead to GT formation [13].

The GTs frequently (90%) present solitary. Multiple GT presentation known as glomangiomatosis is seen mostly in children and is associated with either autosomal dominant inherited mutation in chromosome 1 or neurofibromatosis type 1 [2, 11]. The lesions in glomangiomatosis may not be painful, unlike isolated glomus tumor cases [14].

The average presenting age of GT is usually between 30 to 50 years old and the tumor starts to become atrophied after the age of 60 [11, 15]. Its prevalence in both genders is nearly similar [16]. However, the GT is more likely to be extra-digital in male patients [1, 17].

The GTs can be found in any location, even in visceral organs. There are reported cases of GT affecting breast [18], thyroid [6, 10], heart [7, 19], trachea [2, 20], lung [8], mediastinum [21, 22], esophagus [5, 23], stomach [24, 25], liver [26], small intestine [9] and colon [27]. Extra-digital cases may be missed frequently as a result of affecting less common sites and their atypical presentation. Similarly, our GT case involving the chest wall at the mid-axillary line had long been missed and maltreated. To the best of our knowledge, only 7 cases of GT with chest wall involvement have been reported in the literature [Table 1]. Most reported chest wall GT, interestingly, are from the east and southeast Asia, suggesting a possible role of genetics in this specific variety of the tumor.

**Table 1:** Reported cases of chest wall glomus tumor in the literature

Study	Patient’s GENDER	Patient’s Age (Y/O)	Duration of symptoms (Y)	Location of mass	Relapse
Temiz et al. [31]	Not reported	Not reported	Not reported	Subcutaneous Sternum	No
Kambhampati et al. [14]	Male	47	2	Subcutaneous Left anterior chest wall	No
Neelaiah et al. [33]	Male	46	2	Subcutaneous Anterior chest wall	Not reported
Tsuruta et al. [34]	Female	19	5	Dorsal side of pectoralis major Right anterior chest wall	No
Yim et al. [35]	Male	41	Not reported	Deep in the chest wall muscles Right lateral chest wall	Yes
Uchiyama et al. [36]	Male	50	10	Right 3rd intercostal space	No
Schneller J (37)	Male	30	10	Multifocal in intercostal spaces Left posterior chest wall (largest one)	Not reported

Van Geertruyden et al. demonstrated that the average time between the beginning of GT’s symptoms and its diagnosis is 10 years [28]. This time was 15 years in our patient.

History of paroxysmal pain, which appears spontaneously or following direct compression and exposure to cold temperature, is the main clue to reach the diagnosis [11]. Upon physical examination, Love's test and Hildreth ischemia test are helpful [29]. In Love's test, pain ensues from blunt point pressure on the lesion, and in the ischemia test, pain is relieved following tourniquet inflation. Love's test has up to 100 percent sensitivity, but is not specific in diagnosing the lesion. However, the Hildreth test has up to 92 percent sensitivity, and up to 100 percent reported specificity in diagnosing the GT [11, 30].

Plain radiography generally does not show any abnormality unless in chronic lesions, in which bony erosion may be seen. Although ultrasonography can help in the diagnosis, the imaging modality of choice is MRI [15]. Like other vascular tumors, typical GTs have low signal on T1-weighted MRI and high signal on T2-weighted and T1 post-gadolinium MRI views [16]. Definitive diagnosis can be established only after histopathologic studies. Upon histologic evaluation of GT, nests of epithelioid cells with eosinophilic cytoplasm and round nuclei are detected. Immunohistochemistry distinguishes the GT from many differential diagnoses like schwannoma. GT's immunohistochemistry staining is positive for smooth muscle actin (SMA), vimentin and CD34 and negative for cytokeratin (CK), desmin and neuroendocrine markers (e.g., S100, chromogranin, and synaptophysin) [2].

Surgical excision remains the management of choice with a 10–30% recurrence rate in the literature [11]. Laser ablation and sclerotherapy are demonstrated to have encouraging results in some cases, such as small and multiple lesions [31]**.**

The GT mostly has a benign nature, and it has less than 1 percent risk of transformation into malignancy [11]. However, deep tumor location, diameter more than 2 cm, atypical mitotic figures, and high nuclear grade with more than five mitotic figures per 50 high power fields are proposed to hint toward a possible malignant variety (i.e., glomangiosarcoma) [2, 32]. Depending on the predominant tumor cells, whether vascular, smooth muscle cells, and glomus cells, the benign lesions may be classified as glomangioma, glomangiomyoma, and glomus tumor proper, respectively [2, 16].

In our case, the histopathologic findings were in favor of glomangioma, and no significant nuclear atypia or mitosis was identified. However, the size and deep location of the tumor overlapped with the malignancy criteria. Close observation of the patient and 19-month follow-up has shown neither metastasis nor relapse.

In conclusion, a glomus tumor may affect any part of the human body and be associated with significant functional impairment. Meanwhile, the tumor is curable with surgical excision in most cases. Therefore, early correct diagnosis is of paramount importance. A high index of suspicion would be needed in order not to miss GT cases.

## Data Availability

Not applicable.

## Abbreviations

GT: Glomus tumor
MRI: Magnetic resonance imaging
